# Incidence and Impact of Refeeding Syndrome in an Internal Medicine and Gastroenterology Ward of an Italian Tertiary Referral Center: A Prospective Cohort Study

**DOI:** 10.3390/nu14071343

**Published:** 2022-03-23

**Authors:** Emanuele Rinninella, Marco D’Angelo, Raffaele Borriello, Tiziano Galasso, Marco Cintoni, Pauline Raoul, Michele Impagnatiello, Brigida Eleonora Annicchiarico, Antonio Gasbarrini, Maria Cristina Mele

**Affiliations:** 1 Dipartimento di Medicina e Chirurgia Traslazionale, Università Cattolica Del Sacro Cuore, Largo F. Vito 1, 00168 Rome, Italy; emanuele.rinninella@unicatt.it (E.R.); mariacristina.mele@unicatt.it (M.C.M.); 2 UOC di Nutrizione Clinica, Dipartimento di Scienze Mediche e Chirurgiche, Fondazione Policlinico Universitario A. Gemelli IRCCS, Largo A. Gemelli 8, 00168 Rome, Italy; marco.cintoni@gmail.com (M.C.); pauline.raoul1@gmail.com (P.R.); 3 Scuola di Specializzazione in Medicina Interna, Università Cattolica Del Sacro Cuore, Largo F. Vito 1, 00168 Rome, Italy; dangelo.marco92@gmail.com (M.D.); raffaeleborr@gmail.com (R.B.); tizianog.1993@gmail.com (T.G.); 4 UOC di Medicina Interna e Gastroenterologia, Dipartimento di Scienze Mediche e Chirurgiche, Fondazione Policlinico Universitario A. Gemelli IRCCS, Largo A. Gemelli 8, 00168 Rome, Italy; michele.impagnatiello@policlinicogemelli.it (M.I.); brigidaeleonora.annicchiarico@policlinicogemelli.it (B.E.A.)

**Keywords:** refeeding, malnutrition, ASPEN criteria, mortality, length of hospital stay, readmission

## Abstract

Background: Refeeding syndrome (RS) is a neglected, potentially fatal syndrome that occurs in malnourished patients undergoing rapid nutritional replenishment after a period of fasting. The American Society for Parenteral and Enteral Nutrition (ASPEN) recently released new criteria for RS risk and diagnosis. Real-life data on its incidence are still limited. Methods: We consecutively enrolled patients admitted to the Internal Medicine and Gastroenterology Unit of our center. The RS risk prevalence and incidence of RS were evaluated according to ASPEN. The length of stay (LOS), mortality, and re-admission rate within 30 days were assessed. Results: Among 203 admitted patients, 98 (48.3%) were at risk of RS; RS occurred in 38 patients (18.7% of the entire cohort). Patients diagnosed with RS had a higher mean LOS (12.5 days ± 7.9) than those who were not diagnosed with RS (7.1 ± 4.2) (*p* < 0.0001). Nine patients (4.4%) died. Body mass index (OR 0.82; 95% CI 0.69–0.97), RS diagnosis (OR 10.1; 95% CI 2.4–42.6), and medical nutritional support within 48 h (OR 0.12; 95% CI 0.02–0.56) were associated with mortality. Conclusions: RS incidence is high among clinical wards, influencing clinical outcomes. Awareness among clinicians is necessary to identify patients at risk and to support those developing this syndrome.

## 1. Introduction

Refeeding syndrome is a potentially fatal complication that occurs in malnourished patients when an excessive amount of nutrients is too rapidly delivered after a prolonged state of fasting, as often happens in hospital settings [1]. It is defined as severe electrolyte and fluid shifts, associated with metabolic abnormalities in malnourished patients undergoing refeeding, whether orally, enterally, or parenterally [2]. Up-to-date data about the incidence of RS remain heterogeneous. A recent systematic review of thirty-five observational studies found a wide range of RS incidence, varying from 0 to 62%, among studies [3].

The risk of developing RS is high after prolonged fasting, leading to a reduction in insulin release and an increase in glucagon secretion. Metabolic inversion from the use of glucose, as a source of energy, to the use of structural proteins and lipid deposits occurs with a reduction in intracellular energy cofactors (vitamins and minerals), in particular, phosphorus, potassium, and magnesium. During refeeding, the presence of nutrients, in particular, carbohydrates, stimulates glycolysis, glycogen synthesis, and the synthesis of lipids and proteins, with increased intracellular retention of water and sodium. This anabolic process requires the presence of cofactors, such as thiamine (vitamin B1) and minerals. This results in a rapid reduction in these cofactors at the intravascular level, reduced elimination of sodium and water in the urine, water retention, and the risk of cardiometabolic, respiratory, and neurological decompensation, eventually leading to death [3,4]. Such a condition often occurs during hospitalization, sometimes causing death if not diagnosed and treated promptly [5].

Recently, the American Society for Parenteral and Enteral Nutrition (ASPEN) convened an inter-professional task force to develop a consensus to identify patients at a high risk of developing RS, to prevent and treat it. This consensus, published in April 2020 [6], defined RS as a metabolic and electrolytic alteration that occurs after the reintroduction of nutrition. This retrospective cohort study aimed to assess the prevalence of RS risk and the incidence of RS according to ASPEN criteria, and its impact on length of stay (LOS), mortality, and re-admissions within 30 days in patients admitted to the Internal Medicine and Gastroenterology Unit.

## 2. Materials and Methods

### 2.1. Study Design and Ethical Committee Approval

This was a single-center observational prospective cohort study.

The study was conducted based on the Declaration of Helsinki and according to Good Clinical Practice guidelines. The study was approved by the Ethical Committee of Fondazione Policlinico A. Gemelli IRCCS—Catholic University of the Sacred Heart (Protocol code 2638/22). All participants signed a consent form recording their agreement to take part in the study and to have the results published. This study was reported according to the STROBE guidelines for cohort studies [7].

### 2.2. Patients

All adult (>18 years old) patients admitted to the Internal Medicine and Gastroenterology Unit at the Fondazione Policlinico Agostino Gemelli IRCCS, Rome, Italy, from March 2021 to January 2022, were prospectively evaluated.

The exclusion criteria were patients already undergoing artificial nutrition during a possible stay in the emergency room, the presence of hypophosphatemia on the day of admission, or refusal to participate in the study.

### 2.3. Protocol Description

#### 2.3.1. Protocol Algorithm

Patients were assessed for risk of RS by physicians of the Internal Medicine and Gastroenterology Unit (B.E.A. and M.I.) upon admission. In patients at risk, a specific evaluation (electrolyte and clinical check) was performed 48 h and 5 days after admission (M.D.A., R.B. and T.G.) to identify patients with RS.

Mortality, LOS, and hospital re-admission within 30 days among enrolled patients were recorded by the analysis of medical records.

#### 2.3.2. Determination of RS Risk

The evaluation of RS risk was performed according to the recently released ASPEN criteria [6].

Patients were considered at “moderate risk” if they met two of the following risk criteria:

- BMI was between 16 and 18.5 kg/m^2^;
- A weight loss of 5% of habitual weight was reported;
- There was no or negligible oral intake for 5–6 days OR < 75% of estimated energy requirement for >7 days during an acute illness or injury OR < 75% of estimated energy requirement for >1 month;
- There were low levels of potassium, phosphorus, magnesium, or normal current levels and recent low levels necessitating minimal or single-dose supplementation;
- There was evidence of moderate subcutaneous fat loss;
- There was evidence of mild or moderate muscle loss;
- In presence of higher-risk comorbidities (moderate disease).

Patients were considered at “significant risk” if they met one of the following risk criteria:

- BMI was <16 kg/m^2^;
- A weight loss of 7.5% in 3 months or >10% in 6 months was reported;
- There was no or negligible oral intake for >7 days OR < 50% of estimated energy requirement for >5 days during an acute illness or injury OR < 50% of estimated energy requirement for >1 month;
- There were moderately/significantly low levels of potassium, phosphorus, magnesium, or minimally low or normal levels and recent low levels necessitating significant or multiple-dose supplementation;
- There was evidence of severe subcutaneous fat loss;
- There was evidence of severe muscle loss;
- In presence of higher-risk comorbidities (severe disease).

#### 2.3.3. Diagnosis of RS

The diagnosis of RS was then confirmed according to the above-mentioned ASPEN criteria [6], which are as follows:

- A decrease in serum phosphorus, potassium, and/or magnesium levels by 10–20% (mild RS), 20–30% (moderate RS), or >30%, and/or organ dysfunction resulting from a decrease in any of these and/or due to thiamin deficiency (severe RS).
- The decrease occurs within 5 days of reinitiating or substantially increasing energy provision.

### 2.4. Outcomes Measures

The primary outcome was the incidence of RS risk according to ASPEN 2020 criteria. The secondary outcomes were the diagnosis of RS after the initiation of nutritional support and its impact on mortality, LOS, and hospital readmission within 30 days.

### 2.5. Sample Size Calculation

According to a recent systematic review, the incidence of RS widely varied from 0% to 62% across the studies [3]. However, a previous study identified an incidence of RS risk up to 54% and an RS diagnosis rate of 8% of patients admitted to an internal medicine department [8]. With a margin of error of 6% and a confidence interval of 95%, between 79 and 158 patients should have been enrolled to intercept the above-mentioned incidences (percentages). Considering a dropout rate of 10%, our sample size was set at 176 patients.

### 2.6. Data Collection and Statistical Analysis

Data were collected using a specific Excel^©^ spreadsheet. Data are shown using descriptive statistical methods. The following measures were used as quantitative variables: minimum, maximum, range, mean and standard deviation. The qualitative variables were summarized in tables of absolute and percentage frequencies. The possible normality of continuous distributions wasexamined by applying the Kolmogorov–Smirnov test.

The primary objective was reached by calculating the cumulative incidence of risk of developing RS in the enrolled patients.

The secondary objectives were reached through the cumulative incidence of overt RS development in the enrolled at-risk patients, and the development of a Cox regression model for the evaluation of mean hospital stay, with log-rank analysis to highlight differences between the groups that develop RS or not. To analyze other secondary outcomes, univariate logistic models were created, obtaining odds ratios (OR).

A *p* < 0.05 was set as statistically significant. All statistical analyses were carried out with STATA (version 13, Stata Corporation; College Station, TX, USA).

## 3. Results

### 3.1. Baseline Patients’ Characteristics

Two hundred and three patients were enrolled during 11 months of observation. The clinical and demographical data are presented in Table 1. The mean age was 66.1 ± 14.1 years; there were 127 male patients (62.6%) and 68.5% of the patients (*n* = 139) were admitted from the emergency department (ED). The mean Charlson’s Comorbidity Index (CCI) was 3.0 ± 2.4.

#### 3.1.1. Nutritional Evaluation

The mean body weight was 71.8 ± 16.3 kg and the mean height was 169.0 ± 8.6 cm, deriving a body mass index (BMI) of 25.0 ± 4.9 kg/m^2^. Upon admission, according to nutritional risk screening (NRS-2002), 70 patients (34.5%) were at risk of malnutrition, while, according to the Malnutrition Universal Screening Tool (MUST), 99 patients (48.7%) had the same risk. Twenty-four patients (11.8%) underwent specialist nutritional evaluation during their hospital stay, and 74 (36.5%) were treated with medical nutrition within 48 h from admission, in particular, 63 (64.3%) with oral nutritional supplementation (ONS) and 13 (13.3%) with parenteral nutrition (PN).

#### 3.1.2. Refeeding Syndrome

**Figure 1:**
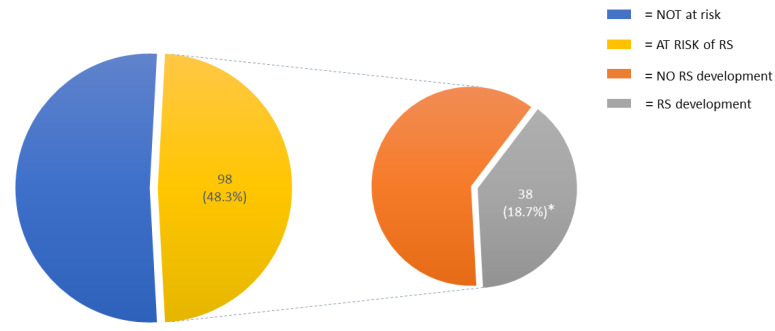
Incidence of RS risk and RS development in the entire cohort. Abbreviations: RS, refeeding syndrome. * = of the entire cohort.

The risk of RS was identified in 98 (48.3%) patients; of these, 44 (21.7%) were at medium risk and 54 (26.6%) were at high risk of developing RS. Thirty-eight patients (18.7% of the entire cohort) developed RS (Figure 1).

Table 2 resumes several data associated with RS development. In particular, blood potassium at 48 h from admission was lower in RS patients (3.9 mmol/L vs. 3.5; OR 0.21; *p* = 0.004), in addition to phosphorus at 48 h (3.3 mg/dL vs. 2.8; OR 0.24; *p* = 0.002) and phosphorus at 5 days (3.4 mg/dL vs. 2.5; OR 0.04; *p* = 0.002).

#### 3.1.3. Length of Hospital Stay

**Figure 2:**
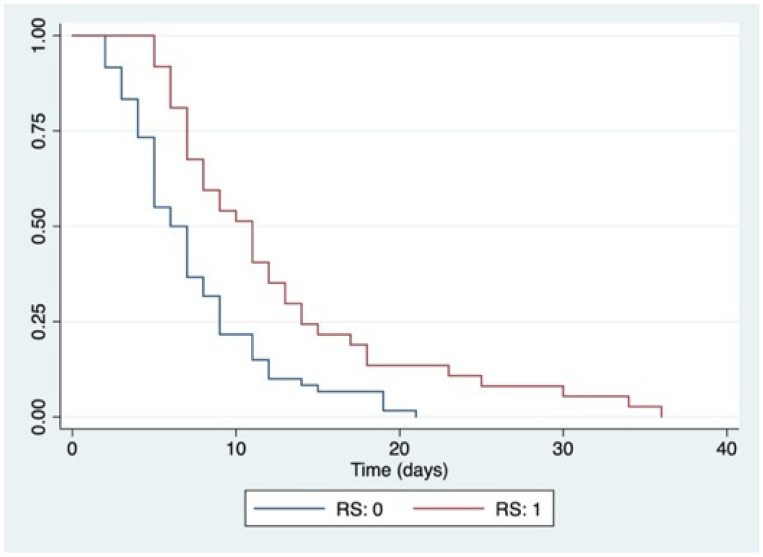
Kaplan–Meier curves for LOS according to RS development. Abbreviations: RS: 0, no development of refeeding syndrome; RS: 1, development of refeeding syndrome; log-rank *p* < 0.0001.

The mean LOS was 8.2 ± 5.8 days. Patients diagnosed with RS had a mean LOS of 12.5 ± 7.9 days. Patients without RS had a mean LOS of 7.1 ± 4.2 days (*p* < 0.0001) (Figure 2).

The principal factors associated with a high LOS are resumed in Table 3. In particular, ER admission (OR 0.38; 95%CI 0.28–0.54), NRS-2002 >3 (OR 0.67; 95% CI 0.49–0.92), MUST ≥2 (OR 0.51; 95% CI 0.37–0.71), RS risk (OR 0.66; 95% CI 0.50–0.88), and RS development (OR 0.45; 95% CI 0.50–0.88) were factors associated with a higher LOS.

#### 3.1.4. In-Hospital Mortality and Hospital Readmission

Nine patients (4.4%) died during hospitalization. BMI (OR 0.82; 95% CI 0.69–0.97), RS development (OR 10.1; 95% CI 2.4–42.6), and medical nutritional support within 48 h from admission (OR 0.12; 95% CI 0.02–0.56) were associated with mortality, even if these associations were different (Table 4). Thirteen patients (6.4%) were re-admitted to hospital within 30 days from discharge; however, none of the tested variables were associated with re-admission (Supplementary Files Table S1).

## 4. Discussion

This observational prospective cohort study consecutively enrolled 203 inpatients admitted to an Internal Medicine and Gastroenterology Ward of an Italian Tertiary Referral Center; most of these patients (68.5%) were admitted from the ED. According to the ASPEN criteria [6], 98 patients (48.3%) were at risk of developing RS and 38 patients (18.7% of the whole cohort) developed RS. During the hospital stay, nine patients died; RS was associated with intra-hospital mortality with an OR of 10.1, which means that the possibility of an inpatient dying from RS or its complications was 10:1, compared to a patient without RS. It is noteworthy to highlight that a higher baseline BMI and the presence of nutritional support were both associated with lower in-hospital mortality. Furthermore, regarding LOS, patients who developed RS had an average hospital stay of five days more than those who did not develop the syndrome (*p* < 0.0001). Interestingly, a longer LOS was associated with a worse nutritional status (NRS-2002 >3 and MUST ≥2) upon ED admission, confirming the role of hospital malnutrition as a predictor of poor clinical outcomes, as previously described [9].

The overall evidence raises the question of how RS is recognized by physicians in clinical practice as a serious clinical challenge. Indeed, emerging literature reports a variable incidence of RS, ranging from 0 to 62% of incidence, depending on the type of studies [3]. Homogeneous data on specific clinical contexts are still limited. With regards to Internal Medicine wards, Kraaijenbrink et al. [8] reported, in 2017, an incidence of RS risk of 54% among 178 admitted patients in the Netherlands, according to the National Institute for Health and Care Excellence (NICE) criteria [10]. In that cohort, only 14 patients (8%) were diagnosed with RS. However, the authors considered the development of severe hypophosphatemia during follow-up, a positive NICE score, and normal phosphate levels upon admission as a hallmark of RS. ASPEN criteria, released in 2020 [6], also include the imbalance of “serum potassium, and/or magnesium, and/or organ dysfunction resulting from a decrease in any of these and/or due to thiamin deficiency”. Such criteria could lead clinicians to a wider identification of RS, otherwise neglected as general electrolyte disturbance. Moreover, early diagnosis is useful to prompt specific nutritional therapy, according to the same ASPEN guidelines [6].

A recent observational cohort study [11] showed a high incidence of refeeding syndrome in patients with total parenteral nutrition in a Brazilian reference university hospital. The authors used the diagnostic criteria of Doig et al. [12] (serum phosphorous levels decreasing by 0.5 mg/dL or lower than 2.0 mg/dL after initiation of TPN, together with a decrease in serum magnesium and/or potassium levels). Data from the electronic medical records of 97 hospitalized patients showed an incidence of RS of 43.3% in patients treated with parenteral nutrition. Interestingly, patients who received standard parenteral nutrition (without the supervision of a team of nutritional specialists) were more likely to develop RS. In turn, our study consecutively evaluated all patients admitted to the ward, and only 13 patients (6.4% of the whole cohort) received parenteral nutrition, while 63 (31%) received oral nutritional supplements within 48 h from admission. As per protocol, the period of observation in our study was limited to the first five days from admission; thus, we could have lacked data after this period. Nevertheless, the incidence of RS remains high.

Of note, in our study, neither nutritional team support nor nutritional supplementation within 48 h (neither oral nor parenteral) influenced the LOS. However, nutritional counseling was delivered in only 24.5% of patients who were considered at risk of developing RS, while nutritional supplementation (within 48 h) was delivered in 75.5% of patients at risk. We did not perform an in-depth analysis to check if nutritional support within 48 h was delivered according to the guidelines. This could be the first limitation of the study, due to its observational nature. Another limitation is the period of follow-up. We decided to limit the observational window of patients who were considered at risk to only five days from admission. We do not have data about the patients who were not considered at risk upon admission. However, collecting data for each patient throughout the entire hospital stay would be unfeasible, so we needed to make a choice.

Furthermore, we decided not to perform a multivariable analysis after the univariable analyses, since almost all the variables significantly associated with the endpoints were collinear. Moreover, there were only a few episodes of mortality and readmissions. Indeed, only 13 patients (6.4%) were re-admitted to hospital within 30 days from discharge. As described in the Supplementary files, we found no significant association with any registered variables. This could be attributed to the absence of a follow-up observation after hospital discharge.

## 5. Conclusions

Our work showed a prevalence of RS risk of 48.3% and an incidence of RS diagnosis of 18.7% in a cohort of 203 patients consecutively admitted to a Department of Internal Medicine and Gastroenterology, according to ASPEN criteria. RS is associated with higher mortality and a higher LOS. Such evidence should raise awareness among clinicians to identify patients at risk of RS early, and to prompt specific nutritional support in patients developing this syndrome.

## Figures and Tables

**Table 1:** Patients’ Characteristics (*n* = 203).

Variables	
Male (*n*, %)	127 (62.6)
Age, in years (mean ± SD)	66.05 ± 14.08
Admission (*n*, %)	
Elective	62 (30.5)
Emergency	139 (68.5)
Other	2 (1)
CCI score (mean ± SD)	3.02 ± 2.43
Body weight, in kg (mean ± SD)	71.76 ± 16.29
Height, in cm (mean ± SD)	169.03 ± 8.56
BMI, in kg/m^2^ (mean ± SD)	25.02 ± 4.88
NRS-2002 > 3	70 (34.5)
MUST score (*n*, %)	
0	73 (36.0)
1	31 (15.3)
≥2	99 (48.7)
Risk of RS (*n*, %)	98 (48.3)
Medium (*n*, %)	44 (21.7)
High (*n*, %)	54 (26.6)
RS diagnosis (*n*, %)	38 (18.7)
Nutrition team support (*n*, %)	24 (11.8) *(24.5) **
Nutritional supplementation within 48 h (*n*, %)	74 (36) * (75.5) **
Oral nutritional supplementation (*n*, %)	63 (31) * (64.3) **
Parenteral nutrition (*n*, %)	13 (6.4%) *(13.26) **
LOS, in days (mean ± SD)	8.24 ± 5.75
In-hospital mortality (*n*, %)	9 (4.4)
Readmission within 30 days (*n*, %)	13 (6.4)

Abbreviations: BMI, body mass index; CCI, Charlson’s Comorbidity Index; CONUT, controlling nutritional status; LOS, length of hospital stay; MUST, Malnutrition Universal Screening Tool; NRS, nutritional risk score; RS, refeeding syndrome; SD, standard deviation. * on the whole cohort; ** on patients at risk of RS.

**Table 2:** Relationships between clinical characteristics and diagnosis of refeeding syndrome (RS) in patients at risk of developing RS (*n* = 98).

	Absence of RS (*n* = 60)	Diagnosis of RS (*n* = 38)	OR (95% CI)	*p*-Value
Male (*n*, %)	35 (58.3)	22 (57.9)	1.02 (0.45–2.31)	0.97
Age in years (mean ± SD)	68.2 ± 12.7	69.9 ± 14.0	1.01 (0.97–1.04)	0.51
ER admission (*n*, %)	52 (88.1)	33 (89.2)	1.11 (0.30–4.08)	0.87
CCI score (mean ± SD)	3.4 ± 2.6	3.2 ± 2.7	0.93 (0.76–1.09)	0.41
Baseline body weight in kg, (mean ± SD)	67.3 ± 15.9	68.8 ± 14.4	1.00 (0.98–1.04)	0.61
Body height in cm, (mean ± SD)	168.4 ± 9.1	167.4 ± 9.6	0.98 (0.94–1.03)	0.57
BMI in kg/m^2^ (mean ± SD)	23.6 ± 4.4	24.6 ± 5.1	1.05 (0.96–1.15)	0.27
NRS-2002 > 3 (*n*, %)	39 (65.0)	22 (57.9)	0.74 (0.32–1.71)	0.48
MUST ≥ 2 (*n*, %)	48 (80.0)	30 (78.9)	0.93 (0.34–2.55)	0.90
High RS Risk (*n*, %)	32 (54.2)	21 (55.3)	1.04 (0.45–2.36)	0.92
Nutrition team support (*n*, %)	14 (25.0)	9 (25.0)	1.01 (0.38–2.62)	0.98
Nutritional supplementation within 48 h (*n*, %)	41 (70.7)	32 (84.2)	2.21 (0.78–6.25)	0.13
Oral nutritional supplementation (*n*, %)	35 (58.3)	27 (71.1)	1.75 (0.73–4.18)	0.20
Parenteral nutrition (*n*, %)	8 (13.3)	4 (10.5)	0.76 (0.21–2.73)	0.68
Na T0, in mmol/L (mean ± SD)	139.2 ± 4.8	139.4 ± 4.1	1.01 (0.92–1.10)	0.81
K T0, in mmol/L (mean ± SD)	3.9 ± 0.4	3.8 ± 0.6	0.83 (0.37–1.86)	0.66
Ca T0, in mg/dL (mean ± SD)	8.9 ± 0.7	9.1 ± 0.7	1.47 (0.81–2.69)	0.206
Albumin T0, in g/L (mean ± SD)	29.5 ± 6.7	28.1 ± 5.9	0.96 (0.90–1.03)	0.28
P T0, in mg/dL (mean ± SD)	3.3 ± 0.6	3.4 ± 0.7	1.16 (0.61–2.199	0.64
Mg T0, in mg/dL (mean ± SD)	2.0 ± 0.3	2.1 ± 0.4	3.42 (0.91–12.81)	0.07
Na 48 h, in mmol/L (mean ± SD)	138.4 ± 4.7	139.0 ± 4.3	1.03 (0.92–1.15)	0.58
K 48 h, in mmol/L (mean ± SD)	3.9 ± 0.4	3.5 ± 0.6	0.21 (0.07–0.61)	**0.004**
P 48 h, in mg/dL (mean ± SD)	3.3 ± 0.7	2.8 ± 0.6	0.24 (0.10–0.60)	**0.002**
Mg 48 h, in mg/dL (mean ± SD)	1.9 ± 0.3	1.9 ± 0.3	0.97 (0.21–4.45)	0.97
Na 5 days, in mmol/L (mean ± SD)	131.5 ± 3.6	138.7 ± 4.1	1.05 (0.91–1.22)	0.49
K 5 days, in mmol/L (mean ± SD)	3.7 ± 0.4	3.6 ± 0.5	0.69 (0.17–2.769	0.60
P 5 days, in mg/dL (mean ± SD)	3.4 ± 0.5	2.5 ± 0.6	0.04 (0.01–0.32)	**0.002**
Mg 5 days, in mg/dL (mean ± SD)	1.8 ± 0.2	1.9 ± 0.4	2.10 (0.26–16.629	0.48

Abbreviations: BMI, body mass index; CCI, Charlson’s Comorbidity Index; CI, confidence interval; MUST, Malnutrition Universal Screening Tool; NRS, nutritional risk score; Na, sodium; K, potassium; P, phosphorus; Mg, magnesium; OR, odds ratio; RS, refeeding syndrome; SD, standard deviation. *p*-values in bold are statistically significant (*p* < 0.05).

**Table 3:** Univariate analyses of risk factors associated with length of hospital stay (*n* = 203).

	HR (95% CI)	*p*-Value
Male	0.89 (0.66–1.19)	0.45
Age	0.99 (0.98–1.01)	0.34
ED admission	0.38 (0.28–0.54)	**<0.0001**
CCI score	1.01 (0.95–1.07)	0.65
Baseline body weight	1.00 (0.99–1.01)	0.12
Body height	1.00 (0.98–1.02)	0.60
Baseline BMI	1.02 (0.99–1.05)	0.08
Baseline NRS-2002 > 3	0.67 (0.49–0.92)	**0.01**
Baseline MUST ≥ 2	0.51 (0.37–0.71)	**<0.0001**
RS risk	0.66 (0.50–0.88)	**0.005**
High RS risk	1.02 (0.67–1.53)	0.92
RS	0.45 (0.31–0.66)	**<0.0001**
Nutritional team support	0.70 (0.43–1.13)	0.14
Nutritional supplementation within 48 h	1.34 (0.84–2.16)	0.21
Oral nutritional supplementation	1.00 (0.74–1.37)	0.96
Parenteral nutrition	0.59 (0.34–1.04)	0.07

Abbreviations: BMI, body mass index; CCI, Charlson’s Comorbidity Index; CI, confidence interval; CONUT, controlling nutritional status; ED, emergency department; HR, hazard ratio; MUST, Malnutrition Universal Screening Tool; NRS, nutritional risk score; RS, refeeding syndrome; SD, standard deviation. *p*-values in bold are statistically significant (*p* < 0.05).

**Table 4:** Univariate analyses of risk factors associated with in-hospital mortality (*n* = 9).

	OR (95% CI)	*p*-Value
Male	0.46 (0.09–2.29)	0.34
Age	1.04 (0.98–1.10)	0.14
ED admission	3.72 (0.46–30.44)	0.22
CCI score	1.12 (0.87–1.45)	0.34
Weight	0.95 (0.89–1.01)	0.06
Height	1.00 (0.92–1.08)	0.91
Baseline BMI	0.82 (0.69–0.97)	**0.02**
Baseline NRS-2002 > 3	2.48 (0.64–9.55)	0.18
Baseline MUST ≥ 2	0.37 (0.05–3.17)	0.37
RS	10.1 (2.4–42.6)	**0.002**
Nutrition team support	0.83 (0.16–4.30)	0.82
Nutritional supplementation within 48 h	0.12 (0.02–0.56)	**0.006**
Oral nutritional supplementation	0.36 (0.03–2.17)	0.21
Parenteral nutrition	4.75 (0.89–25.6)	0.07

Abbreviations: BMI, body mass index; CCI, Charlson’s Comorbidity Index; CI, confidence interval; CONUT, controlling nutritional status; ED, emergency department; MUST, Malnutrition Universal Screening Tool; NRS, nutritional risk score; OR, odds ratio; RS, refeeding syndrome; SD, standard deviation. *p*-values in bold are statistically significant (*p* < 0.05).

## Data Availability

The data presented in this study are available on request from the corresponding author for any academic use upon citation of this article. The data are not publicly available due to privacy and permission restricted to the publication of this article only.

## Supplementary Materials

The following supporting information can be downloaded at: https://www.mdpi.com/article/10.3390/nu14071343/s1, Table S1: Univariate analyses of risk factors associated with readmission (*n* = 13).
