# Supplementary material for: Incidence and Impact of Refeeding Syndrome in an Internal Medicine and Gastroenterology Ward of an Italian Tertiary Referral Center: A Prospective Cohort Study
Source: Nutrients. 2022 Mar 23;14(7):1343. doi: 10.3390/nu14071343 (PMC9002385; doi:10.3390/nu14071343)
Supplement: Supplementary file 1 [file nutrients-14-01343-s001.zip › nutrients-1641236-supplementary.pdf]

Supplementary files

Table S1 . Univariate analyses of risk factors associated with readmission n = 13)

	OR (95% CI)	p-value
Male	1.47 (0.47-4.54)	0.50
Age	0.97 (0.94-1.02)	0.29
ER admission	5.24 (0.66-41.53)	0.11
CCI score	1.11 (0.90-1.38)	0.31
RS-related comorbidity	1.21 (0.38-3.85)	0.74
Weight	1.00 (0.97-1.04)	0.79
Height	0.99 (0.93-1.07)	0.98
Baseline BMI	1.00 (0.89-1.12)	0.93
Baseline NRS-2002 > 3	0.55 (0.14-2.07)	0.37
Baseline MUST	0.75 (0.13-4.03)	0.74
RS risk	1.78 (0.56-5.63)	0.32
High RS Risk	1.42 (0.32-6.32)	0.64
RS	2.04 (0.59-7.01)	0.26
Nutrition Team Support	0.72 (0.14-3.63)	0.69
Nutritional Supplementation Within 48 h	1.15 (0.22-5.94)	0.86
Oral Nutritional Supplementation	2.01 (0.64-6.21)	0.23
Parenteral Nutrition	1.23 (0.15-8.31)	0.85

Abbreviations: BMI, body mass index; CCI, charlson comorbidity index; CI, confidence interval; CONUT, controlling nutritional status; MUST, Malnutrition Universal Screening Tool; NRS, nu-tritional risk score; OR, odds ratio; RS, refeeding syndrome; SD, standard deviation. P-values in bold are statistically significant ($p < 0.05$).
